# COVID-19 and Children’s Well-Being: A Rapid Research Agenda

**DOI:** 10.1007/s10995-021-03207-2

**Published:** 2021-08-24

**Authors:** Rebecca N. Dudovitz, Shirley Russ, Mary Berghaus, Iheoma U. Iruka, Jessica DiBari, Dana M. Foney, Michael Kogan, Neal Halfon

**Affiliations:** 1 Department of Pediatrics, David Geffen School of Medicine, UCLA, Los Angeles, CA USA; 2 UCLA Children’s Discovery and Innovation Institute, Los Angeles, CA USA; 3 UCLA Center for Healthier Children, Families and Communities, 10960 Wilshire Boulevard, Suite 960, Los Angeles, CA 90024 USA; 4 University of North Carolina At Chapel Hill, Chapel Hill, NC USA; 5 US Department of Health and Human Services, Maternal and Child Health Bureau, Health Resources and Services Administration, Rockville, MD USA; 6 Department of Health Policy and Management, Fielding School of Public Health, University of California Los Angeles, Los Angeles, CA USA; 7 Department of Public Policy, Luskin School of Public Affairs, UCLA, Los Angeles, CA USA

**Keywords:** COVID-19, Mental health, Child, Health equity, Research co-design

## Abstract

**Purpose:**

Understanding the full impact of COVID-19 on U.S. children, families, and communities is critical to (a) document the scope of the problem, (b) identify solutions to mitigate harm, and (c) build more resilient response systems. We sought to develop a research agenda to understand the short- and long-term mechanisms and impacts of the COVID-19 pandemic on children’s healthy development, with the goal of devising and ultimately testing interventions to respond to urgent needs and prepare for future pandemics.

**Description:**

The Life Course Intervention Research Network facilitated a series of virtual meetings that included members of 10 Maternal and Child Health (MCH) research programs, their research and implementation partners, as well as family and community representatives, to develop an MCH COVID-19 Research Agenda. Stakeholders from academia, clinical practice, nonprofit organizations, and family advocates participated in four meetings, with 30–35 participants at each meeting.

**Assessment:**

Investigating the impacts of COVID-19 on children’s mental health and ways to address them emerged as the highest research priority, followed by studying resilience at individual and community levels; identifying and mitigating the disparate negative effects of the pandemic on children and families of color, prioritizing community-based research partnerships, and strengthening local, state and national measurement systems to monitor children’s well-being during a national crisis.

**Conclusion:**

Enacting this research agenda will require engaging the community, especially youth, as equal partners in research co-design processes; centering anti-racist perspectives; adopting a “strengths-based” approach; and integrating young researchers who identify as Black, Indigenous, and People of Color (BIPOC). New collaborative funding models and investments in data infrastructure are also needed.

## Significance

*What is already known on this subject?* Children rarely experience severe physical effects from COVID-19, but larger systemic and social disruptions threaten their longer-term health development and well-being.

*What this study adds?* This research agenda identifies future priorities including the mental health impacts of the pandemic on children at different developmental stages, effective strategies for response and for building resilience at individual and community levels, and the integration of BIPOC researchers and communities in strengths-based creative co-design of interventions to improve health equity. New COVID-specific funding streams, collaborative research platforms, and stronger data systems measuring children’s well-being will facilitate these studies.

## Purpose

COVID-19 and the resultant economic standstill have delivered a significant shock to how the United States functions with reverberations across governance, commerce, healthcare, and education, and at multiple levels including individual, family, community, and the entire ecosystem (US Govt, 2020; US Department of Health and Human Services, 2020). Although children typically do not experience severe physical effects from the virus (Viner et al., 2020), at least 300 children in the U.S. have died (American Academy of Pediatrics and Children’s Hospital Association, 2021) and 4000 have been seriously affected by Multisystem Inflammatory Syndrome in Children (MIS-C) (CDC, 2021). In addition, the long-term effects of even mild or asymptomatic COVID-19 infection are not yet certain (Yelin et al., 2020). Similarly, the effects of contracting COVID-19 in the periconception period or during pregnancy are not well understood for either mother or child (Juan et al., 2020; Zaigham & Andersson, 2020). Although early reports suggest that risks of transmission to the fetus are low (Chen et al., 2020; Schwartz, 2020), COVID-19 infection is associated with increased morbidity and mortality in pregnant women, as well as increased risk for preterm birth (Villar et al., 2021). In addition to the direct physical effects of COVID-19 infection, children and families potentially face acute and long-term threats to their health and well-being from the larger systemic and social disruptions resulting from society’s response to the pandemic (Douglas et al., 2020). Although born out of necessity to reduce spread of infection, these dramatic changes have resulted in interrupted schooling (Winthrop, 2020), separation from extended family members (McAdams, 2020), reduced interactions with friends and peers, and parental job changes and losses. These secondary effects may not be as easily measurable, but have the potential to “run deep”, with latent effects that may cause significant harm over the lifespan.

In addition, the pandemic has highlighted long-standing health disparities with individuals identifying as Black, Indigenous, and People of Color (BIPOC) bearing a disproportionate burden of disease, hospitalization, and mortality (Goyal, 2020) Vahidy et al., 2020; Karaca-Mandic et al., 2020). BIPOC families and children, and those from poorer economic circumstances, are also at higher risk from the secondary social and economic impacts of the pandemic. Although the mechanisms underlying these disparities are not fully understood, we do know that people living in low-income and/or BIPOC communities frequently face a syndemic confluence of risks that accumulate and interact over time, predisposing them to carrying an increased allostatic load (Lemke, 2020, Myers, 2020). This increases vulnerability to a range of both acute and chronic diseases which, operating across the life course, could be contributing to the observed disparate impacts of the pandemic.

History suggests that COVID-19 is not likely to be the last pandemic or shock of this kind (Murdoch & French, 2020). Understanding the full impact of COVID-19 on children, families, and communities is critical in order to (a) describe the scope of the problem, (b) identify solutions that can mitigate harm both immediately and in the long-term, and (c) determine ways to build more resilient systems. For these reasons, we sought to develop a research agenda to understand the short- and long-term mechanisms and impacts of the COVID-19 pandemic on children’s healthy development, with the goal of devising and ultimately testing interventions to respond to urgent needs and prepare for future pandemics. Mindful that other groups are addressing the direct biomedical effects of the virus, we chose to focus this research agenda on the secondary impacts of the pandemic on children’s developmental pathways and well-being. Adopting a Life Course Health Development framework (LCHD) (Halfon & Forrest, 2017; Halfon et al., 2014), we sought to emphasize the need to study factors with the greatest potential to shift health trajectories and long-term outcomes. We also aim to include a focus on factors supporting the resilience of parents, children, families and communities in the presence of high risks or adverse circumstances. Although the resulting COVID-19 research agenda represents the synthesis of our thinking 4–6 months after the start of the pandemic in the US, we believe the critical research questions and suggested approaches identified are applicable to the global community seeking to support the well-being of all children and prepare for future pandemics.

## Description

### Recruitment

The Life Course Intervention Research Network (LCIRN) is one of 12 research networks funded by the U.S.’s Health Resources and Services Administration (HRSA)’s Maternal and Child Health Bureau (MCHB). The LCRIN was established in 2018 to bring together a growing and generative Life Course Intervention (LCI) research community, including scientists, methodologists, practitioners, and family advocates in a collaborative research network focused on conducting and disseminating intervention research to promote health and well-being throughout the life course. The LCIRN coordinated the development of this research agenda in collaboration with the MCH Research Networks, engaging leaders and researchers from each network (i.e. a network of networks) as well as additional thought-leaders in the process. To identify participants, MCHB connected the LCIRN leadership to the directors of all 12 funded research networks. The directors of each network chose who should represent them at the meetings. Each entity was asked to send at least 1 representative but could send more. Two additional entities were invited to add additional expertise in family engagement (Family Voices, which contributes to multiple MCHB networks), and child well-being research (National Academies of Science).

### Participants

In total, 46 representatives from ten networks, as well as their research and implementation partners, participated in at least one of a series of four meetings held between June and October 2020 to develop this collaborative agenda, with 30–35 participants at each meeting. Participants represented a broad range of disciplines—medicine, social work, health policy, public health, occupational therapy, and developmental psychology—and included clinical practitioners, researchers, and family advocates. Participants came from more than 20 institutions across the United States, representing groups working across all stages of the MCH life course, with expertise in prevalent child health challenges including autism, obesity, and mental health. A list of participating research networks and the institutional affiliations of their representatives are given in Table 1.

**Table 1:** Maternal and Child Health COVID-19 Research Agenda Setting Participants

Research entity	Institutions
Adolescent and Young Adult Health Network (AYAH)	University of California San Francisco
Autism Longitudinal Data Project Network (ALDP)	Boston University Johns Hopkins University
Autism Transitions Research Project Network (ATRP)	Drexel University Mathematica
Bridging the Word Gap Network (BWG-RN)	University of Kansas
Developmental Behavioral Pediatrics Research Network (DBPnet)	Children’s Hospital of Philadelphia
Healthy Weight Research Network for Children with Autism Spectrum Disorder and Other Developmental Disabilities Network (HWRN)	University of Massachusetts
Life Course Intervention Research Network (LCIRN)	Brown University School of Public Health Center for the Study of Social Policy Johns Hopkins University Tufts University Turnaround for Children UNC-Chapel Hill University of California Berkeley University of California Los Angeles University of Minnesota University of Puget Sound Vanderbilt University Medical Center,
Maternal and Child Health Measurement Research Network (MCH MRN	Rutgers University
Pediatric Emergency Care Applied Research Network (PECARN)	Brown University
Pediatric Research in Office Settings Network (PROS)	Children’s Hospital of Philadelphia
Health Resources and Services Administration, Maternal and Child Health Bureau	N/A
Family Voices	N/A
National Academies of Science	N/A

### Meeting Structure and Approach

The LCIRN hosted a series of four virtual meetings, using interactive technologies to facilitate active collaboration. Participants brainstormed, revised, and refined research questions and project ideas during and in between meetings, which incorporated a family panel and a presentation on anti-racist research approaches to highlight perspectives that are traditionally under-represented in academic research communities.

### Meeting Goals and Focus Areas

As stated above, the overarching meeting goal was to develop a research agenda to understand the short and long-term mechanisms and impacts of the COVID-19 pandemic on children’s healthy development, which led to the following specific objectives for each of the four meetings:

1. To identify the current and future needs of MCH populations most affected by COVID-19;
2. To identify and assess current MCH research response already underway;
3. To identify and develop collaborative projects to address those MCH needs in the short and long term; and
4. To identify potential funding and support strategies

We sought to address the secondary effects of the pandemic on children’s healthy development across life course stages in the context of their developmental ecosystem including individual, family, schools, community, and service systems levels.

The UCLA Institutional Review Board (IRB) was consulted and an IRB waiver or approval was not required for this agenda-setting process. The work was conducted in accordance with all prevailing ethical principles. A synthesis of the outputs from the three meetings is presented below.

### Assessment

At the start of our research agenda-setting process, there were very few publications on the impact of the pandemic on children and families, but new information emerges daily. A literature scan was continuously updated throughout our process, and provided for and interpreted by the group with a focus on issues most relevant to the long-term health of parents and children. LCIRN staff conducted the scan prior to each meting by searching pubmed and medline with relevant terms such as "child," "youth," “pregnancy,” “school,” “family,” and “covid” to highlight areas of emerging concern and collate this for meeting participants for the purpose of grounding discussion in a common understanding of the literature.

In addition to the literature scan, we conducted an environmental scan with meeting participants to identify emerging information about the pandemic’s impact on children and families that may not yet be captured in the published literature. Each person’s impression of the pandemic, based on their observations, lived experience, emerging research and professional viewpoints, was summarized and presented back to the group for feedback and revision. COVID-19-related trends were categorized as either threats/challenges or supports/opportunities for achieving children’s well-being (Table 2). For example, the pandemic has precipitated innovations in and expansions to virtual service provision for physical and mental health care, and education. Changes which had hitherto been slow to evolve such as reimbursement for telehealth, and teaching of whole classes via video-conferencing have been ushered in virtually overnight. If continued, trends such as these could overcome longstanding geographic or logistical barriers to accessing in-person services.

**Table 2:** COVID-19-related Threats/Challenges or Supports/Opportunities for Children's Health and Well-being

Ecosystem level	Threats to children’s health and well-being	Supports/opportunities to foster child resilience to COVID-19 impacts
Individual	• Challenges for adolescents interacting with peers/ romantic relationships • Challenges interacting with parents working from home- parental preoccupation • Challenges meeting basic needs e.g. food scarcity/ access impacting well-being and behavior • COVID-19 impact could lead to disrupted developmental trajectories affecting health, mental health and wellbeing • Decrease in adolescents’ sense of purpose and belonging • Decreased connectedness to caring adults outside the home • Decreased job prospects for adolescents, increased unemployment • Delayed diagnosis of health and developmental issues • Impact of mask wearing on communication and language acquisition for young children • Increased screen time affecting brain architecture and growth • Increased time on social media with potential for cyber-bullying, negative effects on self-esteem • Potential for stress of the pandemic and secondary changes to negatively impact child and youth mental health in the short term	• Decrease in social anxiety for susceptible children with social distancing and virtual learning • Increased awareness of public health • Increased awareness of the importance of getting health information from reputable sources • Increased interest in building resilience • Parents spending more time with children detecting developmental and behavioral challenges earlier • Some children with Intellectual Development Disorder might be faring better at home, being calmer with fewer behavioral outbursts
Family	• Decreased access to non-custodial parents due to restricted movements • Economic stress impacting family well-being and ability to meet basic needs • Increased social isolation of families especially those already isolated due e.g. to parental mental health issues • Lack of contact with grandparents and extended families • Possible increased family discord • Potential for increased domestic violence/intimate partner violence/child maltreatment • Potential for increased parental stress, anxiety, isolation and poor mental health	• Increased focus on building family and child resilience, recognizing the central role of safe and connected relationships leading to the capacity to thrive amid adversity • Increased interest in home cooking and nutritious meal plans • Interest in providing families with additional social, psychological and instrumental supports • Interest in providing supports in new and innovative ways that can be sustained post COVID-19 • Parents and children able to spend more time at home with potential for more positive interactions with and strengthened bonds. Could be a sustained trend if there is an increase in working from home
Schools	• Challenges for young children in understanding masking and social distancing • Disruption of special education services for children with IEPs • Lack of data on children’s well-being either in-school or distance learning to guide best practice • Preschools and child -care centers closing due to lack of funds • Schools not able to provide childcare, food, other social services • Challenges to higher-education systems in meeting increased mental health needs of those transitioning to adulthood	• Schools can support psycho-social development and well-being via distance learning • Schools are finding new ways to educate children regardless of location • School districts and some principals and superintendents are recognizing there is a lack of social-emotional learning in schools, resulting in an opportunity to make great changes • Schools open to re-structuring to better meet the holistic needs of students
Community	• Collapse of non-profit safety net providers • Decrease in city, county and state revenue could impact social and family services • Impact of loss of community activities such as religious services, concerts, team sports • Increased individualism and loss of social cohesion • Lack of awareness of how to meet the challenges with building relationships, reducing stress, gaining skills leading to a culture of hopelessness • Misinformation about the virus • Potential for increased homelessness • Reduced trust in governments and institutions • Uncertainties over the future workplace and how to prepare for it	• Cities are recognizing the importance of investing in early childhood to support well-being • Increased appreciation for nature/ greenspace and interest in home-based activities e.g. food growing, gardening • Openness to youth engagement/participatory research with benefits both for the youth participants and the community
Systems	• Disparities in access to services • Economic recession • Insufficient support for unemployed	• Increased interest in using data to inform practice in health and education • Increased knowledge of the importance of inclusive early childhood programs • Interest in providing services and supports in new and more innovative ways that can be sustained post COVID-19 • Openness to perceiving both the pandemic and racism as public health crises. Rich foundation of conceptual models, research, and new and emerging policy and payment structures that could catalyze lasting positive change • Potential to increase health access for all through telemedicine • Potential to provide sufficient resources to pull people out of spiral of poverty (e.g., basic income, unemployment insurance, health care, etc.) • Willingness to advance virtual and other creative ways to reach families for all services

### Research Questions

With these threats and opportunities in mind, participants went through an iterative process to develop a set of research priorities. Using an LCHD framing, in which health is regarded as a dynamic process that develops over time, being influenced by a wide range of genetic, epigenetic, biological, psychological and social factors, the group focused on those aspects of the pandemic and our response to it that would have the greatest potential to impact the development of children’s well-being over the long-term.. Participants considered a matrix of both life course stages and ecosystem levels (see Appendix Fig. 2). These life stages included prenatal, neonatal, early childhood, middle childhood, adolescence, and young adulthood while the ecosystem levels included the individual, family, community, systems, and policy levels. In a preliminary prioritization exercise, the intersection points with the highest priority for study were identified as early childhood at the family and systems level, and adolescence at the community and systems level.

However, most questions proved to be applicable across all life stages, albeit in different ways, while reciprocal connections across ecosystem levels meant that many questions also cut across level divisions. The group felt this finding emphasized the need for a systems-based approach to studies designed to understand the impact of a population-wide event such as the pandemic.

Table 3 gives a summary of the resulting overarching research questions at each ecosystem level. Each of the major areas of questioning has an agenda for further subdivisions. These more specific research questions at the Individual (Appendix Table 4), Family (Appendix Table 5), Schools (Appendix Table 6), Community (Appendix Table 7) and Systems (Appendix Table 8) levels can be found in the appendix. For example, the sub-questions at the individual level were divided into 5 areas of child development: physical health, mental health, resilience, children with special healthcare needs, and adolescents. The sub-questions at the family level were divided into sections focused on meeting needs, family functioning, and special concerns. At the community level, sub-question sections included disparities, youth/civic engagement, homelessness, and community resources, while at the school level, sub-questions were grouped into remote learning, re-opening, and children with special healthcare needs. Finally, at the systems level, sub-questions were divided into the areas of healthcare, childcare, and measurement.

**Table 3:** Overarching Research Questions

Ecosystem level	Research questions
Individual	• What are the long-term physical impacts of COVID-19 on children’s physical health, mental health, and resilience? • What are the mechanisms through which differences in susceptibility to COVID-19 are operating? • How can they be addressed? • What are the long-term impacts of the pandemic response (i.e. shut down of schools, economic collapse, etc.) on children’s mental health and developmental trajectories? • How does COVID-19 affect developmental and life transitions, particularly for adolescents and the transition to adulthood? • How do we promote health and well-being and provide support to children and adolescents? How can we use new technologies for health promotion? • What are the special challenges/risks/opportunities for children with special healthcare needs? • What makes some children more resilient and how do we build resilience against future threats? • How do we engage parents, children and youth in the research process to promote their mental health, sense of self, agency and positive health development and well-being?
Family	• How is COVID-19 impacting family functioning and development? How does this differ for special populations, such as immigrant families, those with non-English-speaking parents, and families and caregivers of children with intellectual and developmental disabilities? • What resources do families need to maintain their health and well-being? Are those resources available and equitably distributed? What are the greatest needs/gaps? Do these differ for families of color? If so, why?
Community	• What are the impacts of COVID-19 on existing disparities (educational, health, economic, homelessness) and how can we support communities to address disparities and mitigate impacts? • How can communities build resilience and engage youth in identifying and testing interventions?
Schools	• What are the short- and long-term impacts of remote learning on children and adolescents, particularly those with special needs/IEPs? • What resources are needed to safely re-open schools and what are the risks and benefits to children and society associated with in-person versus distance learning? • How can we use this opportunity to transform school culture to focus on whole-child development?
Systems	• How do we meet/support immediate mental health, physical health, and childcare needs? • How do we build systems that will be more responsive and resilient to future pandemics/other threats? • What strategies might be needed to adjust the federal Medicaid match and advance tiered/bundled payments based on the whole child/family needs considering physical, mental, social and relational health risk? • How can we build or adapt measurement systems to study the impact of the pandemic on children and families?

Prior to the first meeting, each participant was invited to submit their top 3 research priorities. This list was honed and added to over the course of the first 3 meetings which result in 18 research areas categorized by ecosystem level. The final meeting focused on prioritizing the research areas. Each attendee had six votes to allocate among a total of 18 research areas. Attendees were given a list of criteria to keep in mind during the prioritization process. The criteria, which were identified during conversations in the first three meetings and refined by the facilitation team, included:

1. High need and impact: Worth pursuing even with high effort because need is high. Addresses the multi-dimensional ecosystem in which children reside. Good use of collaborative resources/assets;
2. Inclusive, anti-racist, collaborative methodologies: High potential to engage disenfranchised communities and produce high-quality data; designed for and with communities most impacted; leverages research as intervention; high potential for sharing data, expanding projects;
3. Responsive and transformative: Can accelerate the well-being of children; responsive, adaptive, flexible, strategic; not just for knowledge’s sake, but to respond to/anticipate challenges, build protective factors and resilience, put in place buffering processes; and
4. Balanced: Contributes to a balanced national research agenda.

**Fig. 1:**
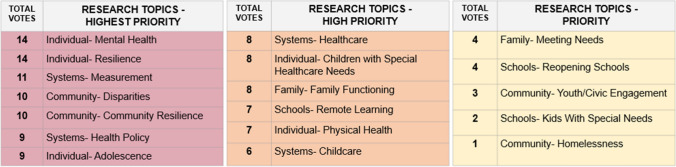
Results of research prioritization polling. Criteria for prioritization included (1) High need and impact (defined as (a)) worth pursuing even with high effort because need is high; (b) addresses the multi-dimensional ecosystem in which children reside; and (c) good use of collaborative resources/assets); (2) Inclusive, anti-racist, collaborative methodologies (defined as (a)) high potential to engage disenfranchised communities and produce high-quality data; (b) designed for and with communities most impacted; (c) leverages research as intervention; and (d) high potential for sharing data, expanding projects); (3) Responsive and transformative (defined as (a)) can accelerate the well-being of children; (b) responsive, adaptive, flexible, strategic; and (c) not just for knowledge’s sake, but to respond to/anticipate challenges, build protective factors and resilience, put in place buffering processes); and (4) Balanced (defined as contributing to a balanced national research agenda). Each attendee had up to 6 votes to allocate. Votes could be used more than once for a single area if desired

Votes could be used more than once for a single area if desired, the results of which are shown in Fig. 1. We acknowledge that this prioritization is reflective of the views of the meeting attendees and that other groups may wish to prioritize differently.

### Recommendations for Enacting the COVID-19 Research Agenda

Given the unique nature of the COVID-19 pandemic and both the challenges and opportunities presented by conducting research during a time of great societal change and upheaval, the group identified recommended approaches for conducting this research. In addition, significant barriers must be overcome to enact this agenda. The final meeting focused on discussing barriers to developing new research collaborations and sharing assets, transforming the research infrastructure, funding, and sustainability.

### Approaches for Future Research

#### Use Anti-Racist Research Methods

An anti-racist research and intervention approach is integral to understanding and intervening on all of the forces and factors leading to COVID-19-related and other health disparities. In 2019, the American Academy of Pediatrics (AAP) issued a policy statement on the impact of racism on child and adolescent health, saying that the “failure to address racism will continue to undermine health equity for all children, adolescents, emerging adults, and their families” (Trent et al., 2019). This will require changes to how we do research, who does research, and how research is funded (Boyd et al., 2020). Potential strategies to address racism and other forms of structural disadvantage that influence research include adopting community-based participatory research (CBPR) methods (Henry Akintobi et al., 2020), including explicit study of the role of racism (Boyd et al., 2020) and structural disadvantage, and addressing structural racism in the research ecosystem itself that limits the questions, theories, methodological approaches, and interpretation of results, as well as the numbers, success, and leadership of BIPOC researchers (Hardeman & Karbeah, 2020). Changes are also needed to funding mechanisms and priorities, as the choice of community-based research topics by BIPOC researchers has been identified as one of the principal reasons they are less likely to be funded (Hoppe et al., 2019). Research must not just document but address inequities, with funding streams that reflect this priority (Boyd et al., 2020). Transparency and exemplary research practices should build trust in the research process, especially in communities where prior unethical and harmful practices have resulted in mistrust of the research community (Scharff et al. 2010).

#### Actively Engage Youth, Families, and Communities

Research studying COVID-19 effects and potential mitigating interventions should be community-driven (Ratneswaren, 2020). CBPR models establish a partnership between the community members and the research team. Using the research agenda questions as a starting point, researchers have an opportunity to work with communities to identify their most pressing questions and needs and co-design the research process. Youth are especially important to engage in this process as they have unique perspectives to assist in identifying key priority areas. For example, youth may adopt new technologies more quickly than older Americans, yet be more vulnerable to mental health threats. Engaging BIPOC youth, in particular, can both expand the perspectives engaged in the research process and strengthen the future research pipeline.

#### Adopt a “Strengths-Based” Approach

Initial publications discussing COVID-19 pandemic impacts on parents and children paint a negative picture, based largely on estimated projections. However, communities can be resilient and have many strengths to build on in times of crisis. Research should include studies of risks and protective factors, as well as propose innovative solutions. Understanding factors that make families and children more resilient can identify interventions to help those more vulnerable. Some changes resulting from the pandemic may have had beneficial effects. Studies should take advantage of the pandemic as a type of “natural experiment” to identify strategies that can be implemented post-pandemic to promote maternal and child health.

#### Focus on Health Equity

The COVID-19 pandemic has highlighted longstanding health disparities among racial/ethnic and social groups. Pre-existing health factors (Rozenfeld et al., 2020) such as diabetes and high blood pressure, conditions increasingly understood to have their roots in childhood, are known risks for COVID-19 adult morbidity and mortality, especially when poorly controlled or under treated (Hamer et al., 2020). There are clear mechanisms through which racism, operating either directly or indirectly, contributes to this picture, for example through increased stress and decreased access to health care. The pandemic has created an opportunity for very detailed research to understand both the reasons for these observed racial/ethnic and social disparities, and to investigate the mechanisms leading to positive health in BIPOC children. Although not all studies have health equity as their main focus, all researchers can suggest ways their research might assist in closing health equity gaps both when applying for funding and publishing results.

#### Search for Transformative Approaches

Schein (1987) argued that it is through change we learn from complex systems. COVID-19 has introduced an unexpected and profound change into our social, educational, health and governance systems acting as a type of “stress test”. We posit that much can be learned about the way these systems function by detailed study of the ways they have adapted using quantitative and qualitative research methods. Studies will need to incorporate pandemic-responsive distanced methods of data collection including use of multi-sector administrative data such as electronic health records. A truly transformative approach will involve transdisciplinary research teams incorporating new methods of collaborative deliberation and “deep listening” reflexivity (Popa et al., 2015), producing a more nuanced understanding of what is happening at the individual, family, and community levels in response to the pandemic. These studies will need to incorporate a strong complex systems perspective, acknowledging the multi-level interactions between pandemic-specific factors and more general risk and protective factors contributing to the full picture.

#### Explore New Partnerships

The pivot to teleworking has fostered increased dialogue among some groups who are geographically distant, and would usually only interact on rare occasions, allowing for new research collaborations. The LCIRN aims to play a convening and linking role in this process, identifying where different groups might have a common interest, introducing them and facilitating initial work efforts, and assisting with identifying funding sources and expertise.

#### Activate New Funding Streams

##### COVID-19 Supplements

Participants recommended compiling a comprehensive list of all ongoing child longitudinal studies that could be supplemented with COVID-19-specific research questions, along with support for methodologists with expertise in child well-being trajectories to explore new ways to combine existing datasets to address questions posed by our agenda. Although open to “Big Data” approaches, participants cautioned about the potential to lose nuance and “sense-making” ability, particularly in relation to BIPOC communities and individual neighborhoods. Longitudinal studies already underway with adult subjects that include detailed family and environmental data could be supplemented by the addition of children and grandchildren as subjects, creating rich two- and even three-generational cohorts. Cross-sectional survey studies such as the Health Services and Resources Administration’s, Maternal and Child Health Bureau’s National Survey of Children’s Health (NSCH) could be supplemented with COVID-19-specific questions on a subset of participants, and with supplemental qualitative interviews about the impact of the pandemic. Further, a longitudinal cohort of children from the 2018 and 2019 NSCHs is planned for 2023 to look at the impact of the pandemic on families and children in the US. Similarly, studies focused on BIPOC and other marginalized populations could be supplemented with COVID-19-specific questions in efforts led by BIPOC scholars focused on understanding the developmental ecosystems operating across the life course leading to resilience or vulnerability to primary and secondary effects of the pandemic.

##### COVID-19 Mini-Grants

Immediate research needs can only be addressed with new sources of near-term support. Traditional funding mechanisms which typically require several months of proposal preparation are poorly suited to support research in response to a crisis. Funders alone or through new collaborations (public, private or public–private partnerships) could provide a series of “mini-grants” allowing researchers to work immediately alongside communities, existing national, state or local programs, and/ or with established research teams, to address this agenda. BIPOC researchers are underrepresented in academia, and minimal gains have been made in recent years despite calls for diversity and inclusion (Heilig et al., 2019). Funding BIPOC scholars and action-based research focused on creative co-design of interventions could be prioritized (Hoppe et al., 2019).

##### Create New Funding Collaboratives

Given this time of economic crisis, funders could collaborate across government departments, private foundations, and public–private partnerships to strengthen data infrastructure, at the national, state, and local levels. In particular, developing funding partnerships across health, education, law, and business sectors can leverage financial resources for greater impact.

#### Support Collaborative Research Platforms

The group suggested a web-based platform to facilitate COVID-related research collaborations, linking researchers and community groups with funding and data sources. A *COVID-19 Research Exchange for Children’s Well-Being* could be supported for a 3–5-year time period to facilitate the implementation of this agenda. Using a networked learning system, a robust research, innovation and learning platform could accelerate knowledge production, translation and dissemination (Britto et al., 2018, Reiller 2017). Facilitating community-academic partnerships can both accelerate scientific knowledge generation and the dissemination and translation of findings into policy and practice. Accelerating this process is critical as the pandemic continues to evolve and communities and policy-makers must make decisions in “real time.”

#### Strengthen Ongoing Data Collection on Children’s Well-Being

We identified a critical need to develop more robust data systems focused on children’s well-being that are embedded in national, state, and local infrastructure. Existing data are limited and insufficiently focused on positive health, leading to the potential for misinterpretation. Our measurement systems typically lack a whole-child view, and often lack strengths-based and positive health measures. Participants urged that deidentified data from electronic health records, which already incorporate a longitudinal component, be utilized wherever possible to study trends and address some of the agenda questions. In addition, such individual health data could be linked with public health and other administrative data sources related to the social determinants of health (e.g. housing, employment, food, education) to provide a more complete picture of child and family well-being.

Participants suggested that each city, county and state document all data currently being collected on children, identified gaps and flaws for assessing children’s health and trends over time. These data integration and expansion efforts could be supported by *Child Well-Being Data Support Grants* for partnerships between researchers and state and local data repositories.

#### Adjust Faculty Advancement Criteria

Existing faculty advancement criteria at universities and research institutions prioritize funding and peer-reviewed publications. The participatory approaches required to tackle this ambitious COVID-19 research agenda require a substantial time commitment by researchers to build community collaborations, establish trust, and work together on study and intervention co-design. Advancement criteria that recognize time spent in these activities would support this agenda. Sadly, even experienced CBPR researchers continue to be evaluated on non-CBPR criteria, leading many to leave academia. Some universities have created two tracks, too often resulting in the CBPR track being regarded as less scientifically rigorous, further limiting progress. Junior researchers who have urged institutions to offer a multitude of pathways for advancement have not been successful. Changes to advancement criteria will require support from the highest levels (Teufel-Shone, 2011). Similarly, as more institutions work to confront racist structures and cultures within academia, BIPOC scholars are disproportionately engaged in this critical justice, equity, diversity, and inclusion work, which is often under-valued according to traditional advancement criteria. Formal compensation and recognition for these efforts can support the academic success of BIPOC researchers.

### Limitations

This research agenda is limited by the views of the stakeholders involved. Although we specifically sought to diversify the perspectives of traditional academic researchers by including families impacted by COVID and an expert in anti-racist research approaches, the resulting research agenda was developed by academic researchers with expertise in maternal-child health and hence, may not reflect the views of youth, parents, families, and communities more broadly. While participants included representatives from 10 of the 12 research networks funded by HRSA’s Maternal Child Health Bureau, spanning all regions of the United States and diverse disciplines, findings may have been different had other representatives from those networks participated or if participants were recruited from networks funded through other agencies. Of note, we sought to develop a U.S. research agenda and the resulting research questions and priorities would likely vary from those of a global research agenda. Finally, given the rapidly changing landscape with respect to knowledge regarding COVID-19 impacts and pandemic recovery, it is possible that findings would have been different, had the research agenda been developed at a different stage in the pandemic. However, many of the questions, priorities, and recommendations are likely to be applicable both now and in the future.

### Conclusion

The COVID-19 pandemic poses real challenges to the aim of closing equity and achievement gaps and ensuring continuous improvement in the health and developmental well-being of all children. Approaching COVID-19 through a life course lens reminds us that there will be both short- and long-term effects, and that interactions between changed individual health attributes and changed family and environmental circumstances will continue to reverberate over time (Settersten et al., 2020). The LCHD model (Halfon et al., 2014) reminds us that health is a developmental process, particularly sensitive to changes at critical life stages. For each research question, there will be distinct lessons learned from studying children at different developmental stages. Not all effects will be apparent in short-term studies, and there is increased need for longitudinal data analysis. Increased and continuous stress over a prolonged period may show few external signs during the pandemic itself, but could result in physiological changes that persist long after the pandemic is over, impacting population health for years to come. The United States represents a paradox in that despite a strong economy we provide poor environments for children, whose health outcomes ranked 36^th^ of 38 countries even prior to COVID-19 (UNICEF Innocenti, 2020). While we do not yet know how the US will rank post-COVID there is clearly no place for complacency. Instead, taking what we learn from studying the response to this shock, and using this knowledge to create a new developmental ecosystem for children could be transformative. The pandemic of 2019–2020 might ultimately be remembered not just for its serious health impacts and social disruptions but as the catalyst for change, stimulating the realization of health equity and re-fashioning systems of care to support optimal health development trajectories in early life and throughout the life course. Historically, lasting innovations have often been prompted by response to a crisis. Creative uses of new technology, and a willingness to work together in new ways, hold promise for novel solutions both to the new threats posed by COVID-19 and to long-standing challenges to the well-being of all children that can result from enacting this research agenda.

For a detailed report, please visit https://lcirn.ucla.edu/covid-19agenda

## Figures and Tables

**Fig. 2:**
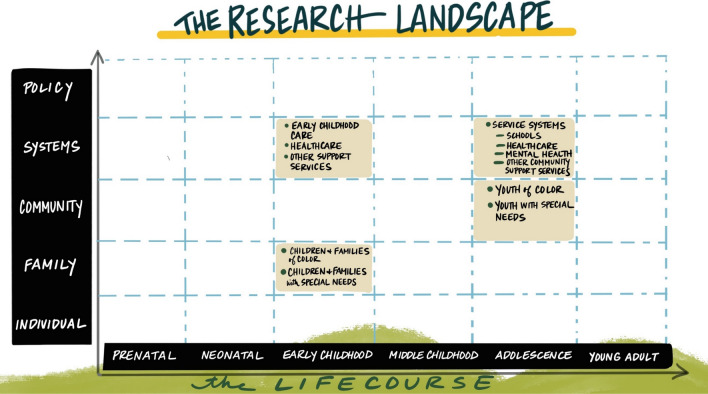
The research landscape: prioritizing life stages and ecosystem levels. Early childhood: children and families of color, children and families with special needs (family level); early childhood care, healthcare, other support services (systems level). Adolescence: youth of color, youth with special needs (community level); service systems: schools, healthcare, mental health, other community support services (systems level). Artwork credit: Giselle Chow for Leapfrog Consulting

## Appendix

See Table 4, 5, 6, 7, 8 and Fig. 2.

**Table 4:** Specific Research Questions at the Individual Level

Physical Health	• COVID-19 Multi-Inflammatory Syndrome in Children (MIS-C) • What are the risk factors for MIS-C? • What are effective treatments for MIS-C? • What are the long-term outcomes for MIS-C? • COVID-19 risk in children • What are the risk and protective factors for the susceptibility and severity of COVID-19? • Are children with pre-existing conditions and children with special health care needs at higher risk for infection or for COVID-19-associated morbidity and mortality? • What are the long-term impacts on children? Does this vary by developmental stage and other child or family characteristics? • What are the effective strategies for mitigating long-term negative impacts of infection on child health and wellbeing? • What are the underlying mechanisms of any racial/ethnic differences in the clinical course of COVID-19? • COVID-19 risk in mothers • Are pregnant women more or less prone to getting COVID-19? • What is the impact on the fetus if the mother contracts COVID-19 at different stages of gestation? • How do infection prevention practices impact babies and mothers (e.g. separation, delayed breastfeeding)? • Physical activity and weight • To what extent has the COVID-19 pandemic given rise to unhealthy weight gain among children? • What impact has the pandemic had on physical fitness in children and adolescents and • how is this related to mental health and social-emotional well-being? Is the impact different for team sports versus individual physical activity? • How has the pandemic impacted the ability of families and caregivers of children with intellectual and developmental disabilities to access opportunities for physical activity and recreation?
Mental Health	• How has the pandemic impacted children’s mental health and development? What disparities exist? • How are mental health and neurodevelopmental trajectories (and educational outcomes) impacted by • Social distancing/isolation? • Changes in personal daily routines? • Increased sources of stress? Personal experience of trauma? • Changes in access to mental health services? The use of telemedicine for mental healthcare delivery?
Resilience	• What are the components and skills needed to meet crises with resilience and how can we build radical resilience for future environmental and social threats? • Where are children thriving and flourishing despite the challenges and what is different for them in terms of family resilience, community supports, orientation/mindsets about the crisis, variations in existing social and relational risks, etc.? • How can we better measure child, family and community resilience? • How can we foster resilience effectively and reliably?
Children with special healthcare needs	• What are the potential risks and/or benefits of full remote learning models for children with ADHD and/or learning disabilities? • What challenges do children with intellectual or developmental disabilities face in engaging in preventive measures (e.g., social distancing, mask wearing)? How has this affected children’s and families’ ability to access opportunities outside of the home? • What is the effectiveness of virtual therapies (behavioral supports, social skills) for children with Autism Spectrum Disorder (ASD)? How has access to services changed and how do these changes impact children’s ability to make developmental gains?
Adolescents	• What interventions and policies can best support adolescents and young adults, given the difficulty in engaging in developmentally appropriate behavior? • How can we engage adolescents and young adults (AYAs) in the research? • How can we use new technologies to reach AYAs in ways that promote health (broadly defined)? • What role and impact can adolescent engagement, activation and inclusion in the development and implementation of social and community solutions play in enabling adolescents to not only contribute but to support their adaptive responses that build enduring developmental capabilities? • Has social distancing had a positive or negative impact on risky health behaviors? • What are the long-term educational and vocational impacts on adolescents who have had suboptimal virtual learning experiences?

**Table 5:** Specific Research Questions at the Family Level

Meeting needs	• What are the top concerns of families of color in relation to the pandemic, and how do they differ from concerns of families of other races/ethnicities? What might this tell us about how our society functions? • How have families' sense of the future changed, and how has that altered parenting behaviors? • What do families think they need and are these resources available but not used or are there new resources needed? • What are the greatest needs for families -are they material, relational? What of the needs are COVID-19 related or just surfaced to our attention due to the crisis?
Family functioning	• How has the pandemic changed guardian/dependent relationships? Have the changes been positive or negative? • What are the changes in role development in families? • What are the impacts on family cohesion?
Special Concerns	• Among immigrant families and those with limited English proficient parents, is COVID-19 accelerating the process of adultification of children and young adults by requiring them to help navigate new technologies and public health messaging as translators? • How has the pandemic impacted the ability of families and caregivers of children with intellectual and developmental disabilities to provide healthy meals?

**Table 6:** Specific Research Questions at the Community Level

Disparities	• How might the pandemic broaden disparities between low-income and higher-income families as many communities shift to virtual education models? • What mitigates health disparities during pregnancy and early childhood?
Youth/civic engagement	• Can young people be engaged in building back better in a way that promotes their mental health, sense of self, agency and positive health development and well-being? • How can youth voices be engaged to address their policy and system needs during the pandemic? • How has the pandemic shaped civic engagement and what are the impacts of civic engagement on health?
Homelessness	• How does the pandemic further affect the ability of youth experiencing homelessness to safely and successfully transition to adulthood? • What can we do to prevent increased homelessness among unaccompanied minors and youth as a result of the pandemic?
Community resources	• What role does religious affiliation play in child outcomes post pandemic? • Identify whether and how community investments in early childhood education, anti-poverty programs, affordable housing and other social determinants of health have mitigated the negative impact of COVID-19 and the pandemic response on children and families

**Table 7:** Specific Research Questions at the School Level

Remote learning	• Strategies • What educational strategies may be associated with better academic and mental health outcomes for adolescents participating in full remote learning models due to COVID-19? • How can schools support psycho-social wellbeing and student engagement via distance learning and what lessons from this experience can be applied long-term to foster relational capacity in schools? • Impacts • What impact has remote learning had on early childhood social behavioral development? How will these impacts affect children and society in the long run? • What is the impact of school disruption on adolescent wellbeing, mental health, social networks, and risky health behaviors? • What can we do to decrease high school drop-out and community college and university disengagement due to the pandemic?
Re-opening	• What resources, policies and practices are needed to safely open schools and what are the risks and benefits to children and society associated with in person versus distance learning? • What are the opportunities for schools to open up safely and with much more emphasis on whole-child learning and development? • How can a networked learning and improvement approach be used to optimize the strategies that schools develop and implement to optimize successful reopening?
Children with special healthcare needs	• What are the potential risks and/or benefits of full remote learning models for adolescents with ADHD and/or learning disabilities? • What is the effectiveness of virtual therapies (behavioral supports, social skills) for children with ASD and other developmental needs?

**Table 8:** Specific Research Questions at the Systems Level

Healthcare	• How can we manage the need for mental health services, given the shortage of mental health providers? E.g., how can we better equip primary care providers? What is feasible? • What levers exist in pediatrics now that we can pull to advance high-quality virtual well-child care using existing resources like the Well Visit Planner, etc.? • What is the acceptability of or barriers to telehealth for developmental-behavioral pediatric care of children with ASD, to providers and parents? • What strategies might be needed to adjust the federal Medicaid match and advance tiered/bundled payments based on the whole child/family needs considering physical, mental, social and relational health risk?
Childcare	• How has the pandemic impacted childcare and early intervention professionals? What supports do they need to address those needs?
Measurement	• How can we conduct more rapid and responsive measurement of the impacts of COVID-19? • What resources can we use to measure collective MCH Network activities addressing COVID-19 and child, family and education intervention and service? • What key metrics are missing at the national, state, local and practice/community level that we can fast track specification and testing for? • How can we collect data from/with/by the people directly involved, including youth?
